# Compensatory classification in spine sagittal malalignment with lumbar degeneration

**DOI:** 10.1186/s12891-023-06310-3

**Published:** 2023-03-27

**Authors:** Yu Wang, Xiang-Yu Li, Wei-Guo Zhu, Cheng-Xin Liu, Chao Kong, Shi-Bao Lu

**Affiliations:** 1 Department of Orthopedics, Xuanwu Hospital, Capital Medical University, No.45 Changchun Street, Xicheng District, Beijing, China; 2 National Clinical Research Center for Geriatric Diseases, Beijing, China

**Keywords:** Spine-pelvic sagittal morphology, Balance, Lumbar degeneration

## Abstract

**Objective:**

To generate a compensatory classification to evaluate sagittal spinal malalignment with lumbar degeneration.

**Methods:**

We included 162 patients with low back pain who underwent full-length spinal radiography in our hospital from August 2019 to October 2021. Using full-length spine X-rays, we measured pelvic tilt (PT), sacral slope (SS), pelvic incidence (PI), thoracic kyphosis (TK), lumbar lordosis (LL), C7 slope (C7S), thoracolumbar kyphosis (TLK), and C7 sagittal vertical axis (SVA). We also recorded the Oswestry Disability Index (ODI) and visual analog scale (VAS). Patients were divided into four groups based on the SRS-Schwab classification and four other groups based on the compensatory classification.

**Results:**

ODI correlated with age, SS, LL, TK, C7-SVA, SRS-Schwab classification, and compensatory classification. Lumbar VAS score correlated with LL, TK, C7-SVA, SRS-Schwab classification, and compensatory classification. Leg VAS score only correlated with LL. Hidden imbalance and imbalance with compensation had more significant PT and larger TK than balance patients. The symptoms of the four compensatory classification groups gradually worsened.

**Conclusion:**

The spinal-pelvic sagittal balance in patients with lumbar degeneration based on pelvic and thoracic compensation can reflect spinal balance and symptoms. This parameter might help evaluate spine sagittal alignment in elderly patients with lumbar degeneration.

## Introduction

Lumbar degenerative disease refers to pathological changes in the lumbar structure, including lumbar intervertebral disk degeneration, facet joint degeneration, ligamentum flavum thickening, and hyperosteogeny due to natural aging and degeneration of the lumbar spine; it causes low back pain and loss lumbar spine stabilization [1, 2].

Lumbar degeneration causes morphological changes, including the loss of lumbar disk height and vertebral slip deformation, leading to reduced LL and kyphosis. Some patients suffer forward movement of the spinal center of gravity and the spine-pelvis sagittal plane imbalance. To prevent aggravation of spinal-pelvic sagittal imbalance, there is often a compensatory mechanism for the spine and pelvis to prevent sagittal spinal imbalance, including pelvic supination, reduction of TK, an increase of cervical lordosis, and other compensation mechanisms [3]. Thus, the sagittal imbalance of the spine occurs during spine degeneration. Garbossa et al. classified sagittal balance of the spine as the balanced spine (SVA < 5 cm), the hidden unbalanced spine (SVA < 5 cm with active pelvic compensation), and the unbalanced spine (SVA > 5 cm) [3].

Until recently, the evaluation of spine sagittal plane balance was based on the C7 sagittal vertical axis (SVA). Most studies found that C7-SVA greater than 4 cm (or 5 cm) was the standard of spine sagittal plane imbalance [4, 5]. Other studies pointed out that C7-SVA was related to symptoms: larger C7-SVA indicated worse symptoms [5, 6]. However, in the elderly population, because of the degenerative changes in spinal morphology, there is no consensus regarding the reasonable range of C7-SVA. With increasing age, the acceptable range of C7-SVA increases [7]. For these reasons, C7-SVA is limited for evaluating the sagittal balance of the spinal pelvis. The evaluation of adult spinal deformity is currently based on the SRS-Schwab classification [7], which includes pelvic incidence (PI)—lumbar lordosis (LL), C7-SVA, and pelvic tilt (PT) to describe the severity of the sagittal deformity spine. The relationship between the SRS-Schwab classification and symptoms needs to be clarified. Therefore, this study aims to determine the correlation between pelvic compensation, thoracic compensation, and symptoms. We proposed a compensatory classification to guide the evaluation of spine sagittal deformity and treatment of sagittal spinal imbalance.

## Methods

### Patient information

This study included patients who underwent full-length spinal radiography at our hospital for low back pain from August 2019 to October 2021. The inclusion criteria were a history of low back pain for more than six months and the ability to maintain a standing posture for a short time. The exclusion criteria were congenital or idiopathic spinal deformity, history of spinal surgery, previous history of thoracolumbar fracture, thoracolumbar kyphosis (TLK) more than 20°, and neck-shoulder pain or upper back pain in the preceding six months.

### Spine-pelvic parameter measurement

**Fig. 1:**
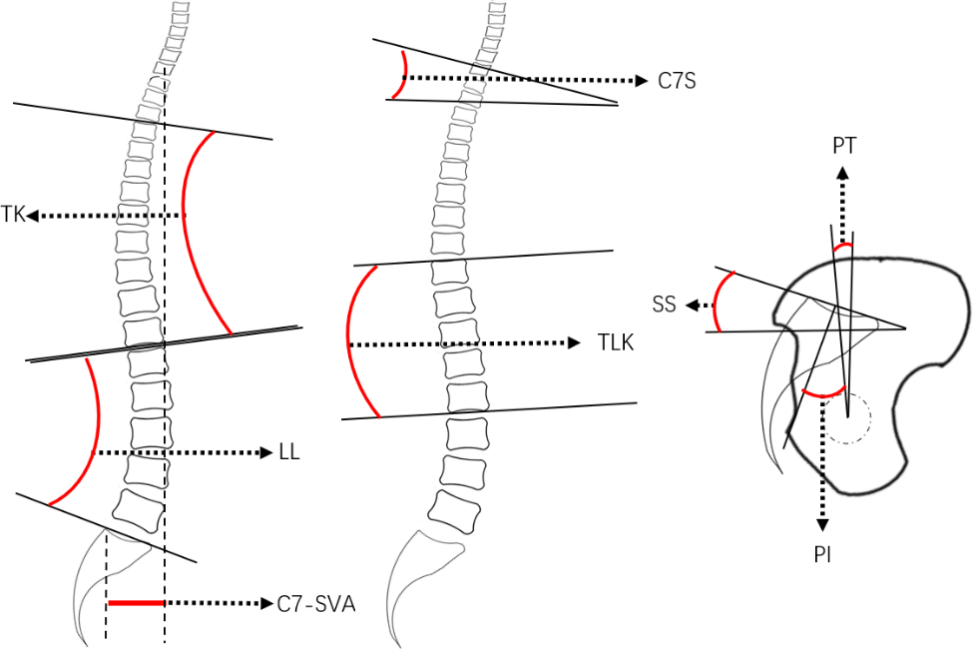
Method to measure spine sagittal alignment. PT: pelvic tilt; SS: sacral slope; PI: pelvic incidence; TK: thoracic kyphosis; LL: lumbar lordosis; C7S: C7 slope; TLK: thoracolumbar kyphosis; C7-SVA: C7 sagittal vertical axis

The patient was required to stand, place the upper limbs in front of the body, look forward, and maintain motionless for two minutes. The measurement parameters and methods were as follows: PT is the angle between the line from the midpoint of the S1 upper endplate and the midpoint of the line connecting the center of the femoral head on both sides vertical line. PI is the angle between the line from the S1 upper endplate’s midpoint to the line’s midpoint connecting the centers of the femoral heads on both sides and the vertical line of the S1 upper endplate. Sacral slope (SS) is the angle between the S1 upper endplate and the horizontal line. LL is the angle between the S1 upper endplate and the L1 upper endplate. TLK is the angle between the L2 lower endplate and the T12 upper endplate. Thoracic kyphosis (TK) is the angle between the T12 lower endplate and the T4 upper endplate. C7 slope (C7S) is the angle between the C7 upper endplate and the horizontal line. C7-SVA is the distance between the plumb line at the center of the C7 vertebral body and the posterior upper corner of S1. The measurement diagram is shown in Fig. 1. All parameters were measured and calculated by two spine surgeons specializing in musculoskeletal disorders with more than five years of experience.

### SRS-Schwab spinal deformity classification

**Fig. 2:**
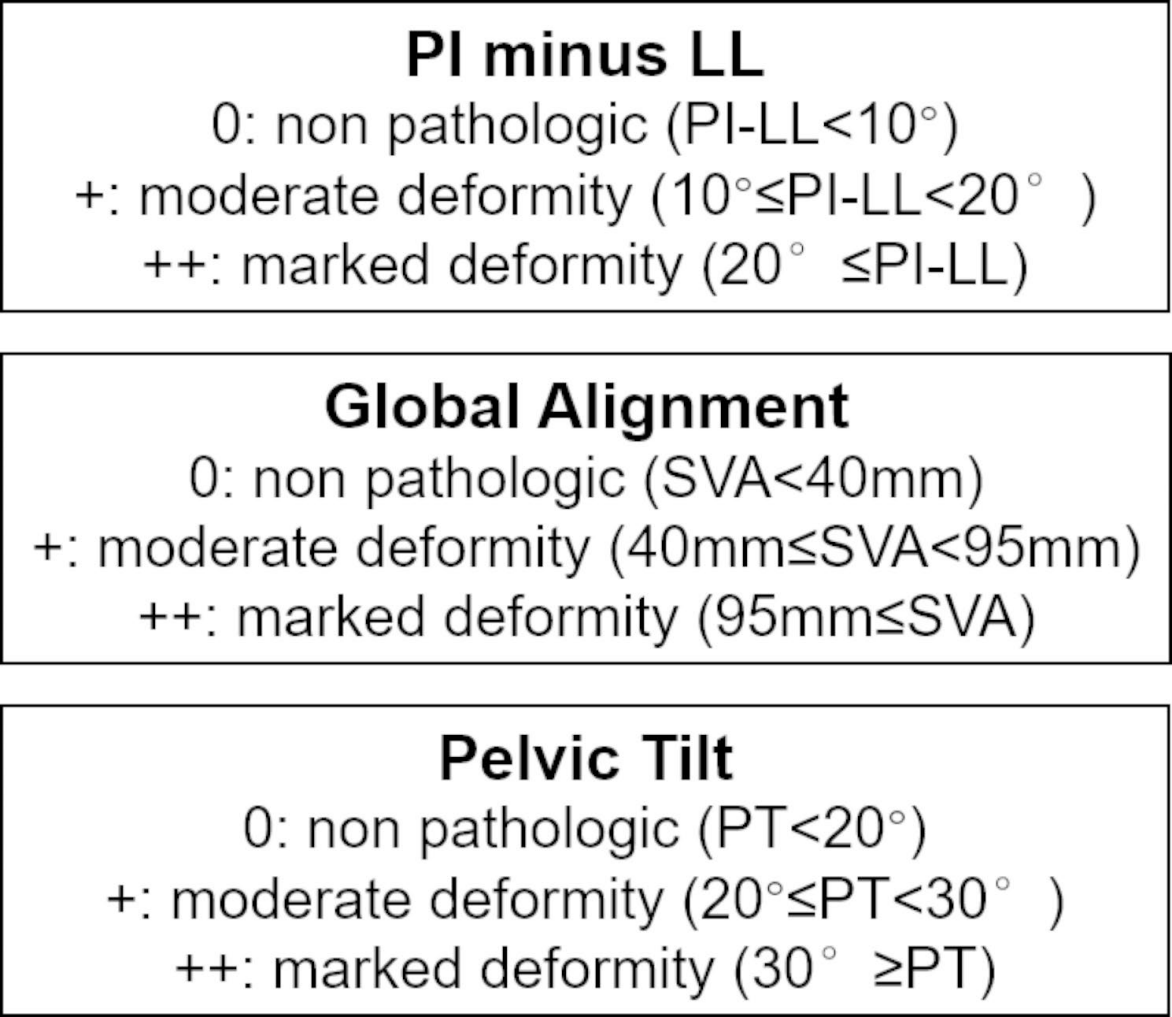
The sagittal spinal modifiers in the SRS-Schwab classification for adult spinal deformity. PT: pelvic tilt; PI: pelvic incidence; LL: lumbar lordosis; SVA: sagittal vertical axis

According to the sagittal spinal modifiers in the SRS-Schwab classification of adult spinal deformity (Fig. 2), patients were categorized as follows: a normal sagittal spinal alignment (Normal) group, comprising patients with standard modifiers in all three sagittal spinopelvic modifiers; a mild sagittal spinal malalignment (Mild) group, comprising patients with at least one sagittal spinopelvic modifier graded as a moderate deformity (+); a moderate sagittal spinal malalignment (Moderate) group, comprising patients with moderate deformity (+) in all three sagittal spinopelvic modifiers; and a severe sagittal spinal malalignment (Severe) group, comprising patients with marked deformity (++) in all three sagittal spinopelvic modifiers.

### Compensatory classification

**Fig. 3:**
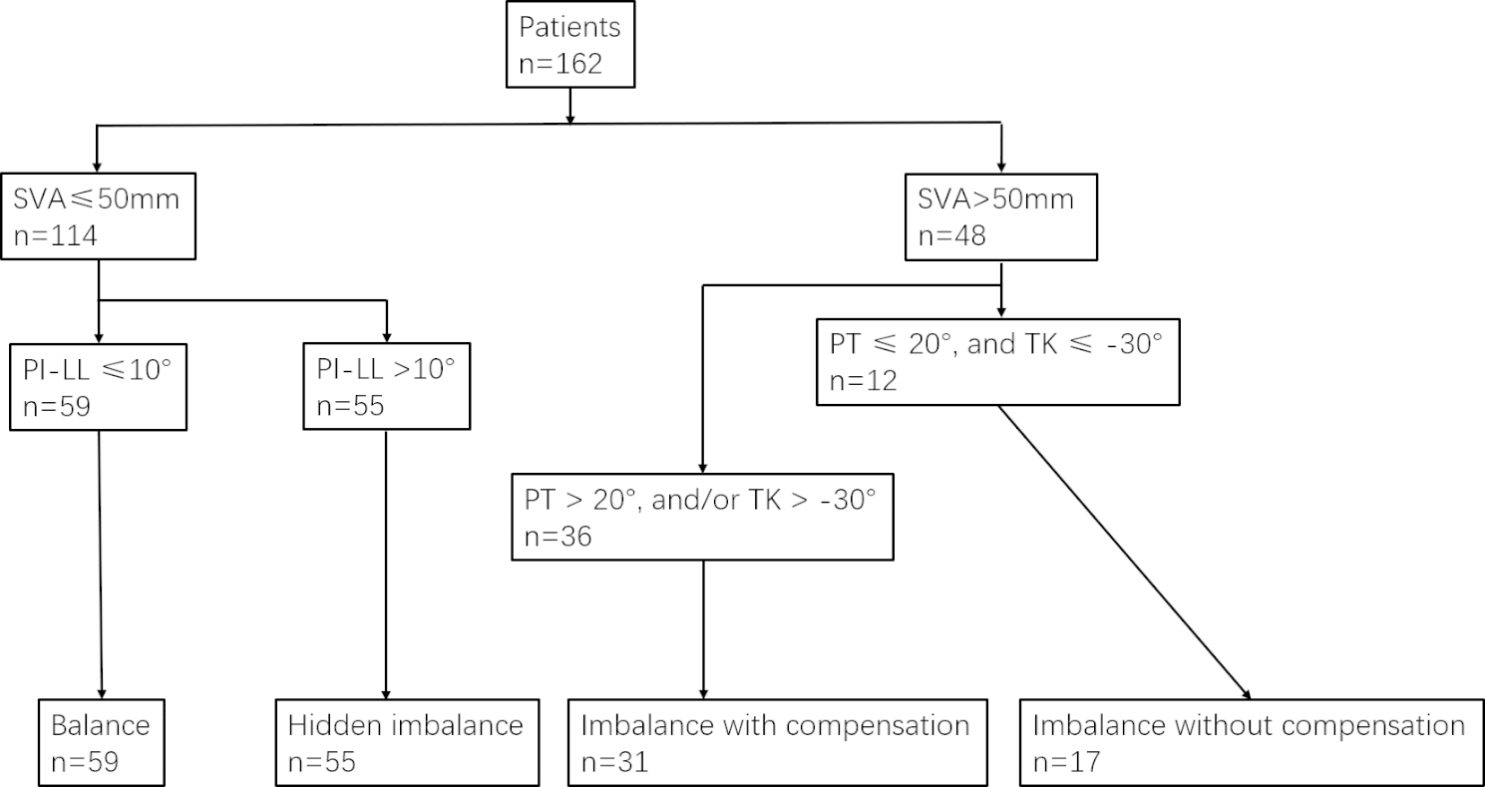
The flowchart of group classification. PT: pelvic tilt; PI: pelvic incidence; TK: thoracic kyphosis; LL: lumbar lordosis; SVA: sagittal vertical axis

According to Li et al., thoracic extension compensation is TK greater than or equal to − 30° [2]. According to the SVA value, patients were divided into a Sagittal balance group (SVA ≤ 50 mm) and an imbalance adult spine deformity (ASD) group (SVA > 50 mm). In the Sagittal balance group, we measured PI-LL. Patients with PI-LL ≤ 10° were classified as Group Balance. Patients with PI-LL > 10° were placed in the Hidden Imbalance group. In the Imbalance ASD group, we measured PT and TK. Patients with PT > 20° or TK > − 30° were classified as the Imbalance with the Compensation group. Patients with PT ≤ 20° and TK ≤ − 30° were classified as the Imbalance without Compensation group (Fig. 3).

### Clinical symptom assessment

Lumbar function was evaluated using the Oswestry Disability Index (ODI), and the degree of low back and lower limb pain was evaluated using a visual analog scale (VAS).

### Data analysis

All collected data were analyzed with IBM SPSS Statistics, version 25.0 (IBM Corp., Armonk, NY, USA). Statistical analysis was performed with the Pearson correlation analysis, Kruskal-Wallis H test, one-way analysis of variance, Tamhane T2 post hoc test, and the Fisher least significant difference post hoc significance test. We performed stepwise regression to identify risk factors for severe symptoms. The results were expressed as the mean value ± standard deviation. A probability (P) value of < 0.05 was considered statistically significant.

## Result

We included 162 patients with low back pain. The average age was 64.86 ± 11.06 years, with 50 males and 112 females. The mean ODI score was 22.33 ± 15.52%. The mean Lumbar VAS score was 4.74 ± 2.13, and the mean Leg VAS score was 2.80 ± 1.85. The sagittal parameters of the spine are displayed in Table 1.

**Table 1:** Patient Characteristics

	Descriptive statistics
Age (years)	64.86 ± 11.06
Gender (M/F)	50/112
PI (°)	50.90 ± 9.02
PT (°)	18.18 ± 8.96
SS (°)	32.75 ± 8.76
LL (°)	35.67 ± 12.67
TLK (°)	-7.28 ± 7.69
TK (°)	-32.75 ± 11.35
C7S (°)	24.50 ± 5.76
C7-SVA (cm)	2.97 ± 4.52
ODI (%)	22.33 ± 15.52
Lumbar VAS	4.74 ± 2.13
Leg VAS	2.80 ± 1.85

PT: pelvic tilt; SS: sacral slope; PI: pelvic incidence; TK: thoracic kyphosis; LL: lumbar lordosis; C7S: C7 slope; TLK: thoracolumbar kyphosis; C7-SVA: C7 sagittal vertical axis; ODI: Oswestry Disability Index; VAS: visual analog scale

Pearson correlation analysis was performed for symptom-related indexes and patient characteristics (Table 2). The ODI correlated with age, SS, LL, TK, C7-SVA, SRS-Schwab classification, and compensatory classification (P = 0.044, P = 0.019, P < 0.001, P = 0.015, P < 0.001, P < 0.001, and P < 0.001, respectively). The Lumbar VAS score correlated with LL, TK, C7-SVA, SRS-Schwab classification, and compensatory classification (p < 0.001, P = 0.002, P < 0.001, P < 0.001, and P < 0.001, respectively). The Leg VAS score only correlated with LL (P = 0.034).

**Table 2:** Pearson correlation analysis for symptom-related indexes and patient characteristics

		ODI	Lumbar VAS	Leg VAS
Age	Pearson coefficient	0.158	0.121	0.147
	P	0.044	0.124	0.062
PI	Pearson coefficient	-0.089	-0.001	-0.153
	P	0.261	0.988	0.052
PT	Pearson coefficient	0.089	0.124	-0.004
	P	0.260	0.116	0.960
SS	Pearson coefficient	-0.184	-0.129	-0.154
	P	0.019	0.102	0.051
LL	Pearson coefficient	-0.371	-0.373	-0.166
	P	< 0.001	< 0.001	0.034
TLK	Pearson coefficient	0.038	0.018	-0.138
	P	0.629	0.818	0.080
TK	Pearson coefficient	0.191	0.243	0.095
	P	0.015	0.002	0.227
C7S	Pearson coefficient	0.062	0.056	0.002
	P	0.435	0.481	0.984
C7-SVA	Pearson coefficient	0.379	0.393	0.135
	P	< 0.001	< 0.001	0.086
SRS-Schwab classification	Pearson coefficient	0.405	0.366	0.060
	P	< 0.001	< 0.001	0.447
Compensatory classification	Pearson coefficient	0.626	0.603	0.021
	P	< 0.001	< 0.001	0.789

*, P < 0.05; **, P < 0.01

PT: pelvic tilt; SS: sacral slope; PI: pelvic incidence; TK: thoracic kyphosis; LL: lumbar lordosis; C7S: C7 slope; TLK: thoracolumbar kyphosis; C7-SVA: C7 sagittal vertical axis; ODI: Oswestry Disability Index; VAS: visual analog scale

We compared demographic data and radiological parameters among the four SRS-Schwab classification groups (Table 3) and found no significant differences in sex, TLK, and Leg VAS (P = 0.766, P = 0.693, and P = 0.524, respectively). However, the four groups showed significant differences in age, PI, PT, SS, LL, TK, C7S, C7-SVA, ODI, and Lumbar VAS (P = 0.06, P < 0.001, P < 0.001, P < 0.001, P < 0.001, P = 0.005, P = 0.008, P < 0.001, P < 0.001, and P < 0.001, respectively). ASD patients were older and had larger PT, smaller SS, less LL, less TK, larger C7S, larger C7-SVA, higher ODI, and higher Lumbar VAS than the normal patients (P < 0.001, P < 0.001, P < 0.001, P < 0.001, P = 0.007, P = 0.001, P < 0.001, P < 0.001, and P = 0.035, respectively).

**Table 3:** Comparison among SRS-Schwab classification groups

	Normal (51)	Mild (81)	Moderate (23)	Severe (7)	p
Age (years)	61.37 ± 11.27!@	65.10 ± 11.29	69.57 ± 8.09!	72.00 ± 5.77@	0.006
Gender (M/F)	16/35	27/54	5/18	2/5	0.766
PI (°)	47.66 ± 7.74!@	50.53 ± 8.92#	57.64 ± 7.50!#	56.59 ± 10.65@	< 0.001
PT (°)	11.34 ± 3.99!@#	18.58 ± 7.62!$%	26.05 ± 5.94@$^	37.49 ± 6.92#%^	< 0.001
SS (°)	36.32 ± 8.08!@#	31.99 ± 8.05!$	31.64 ± 7.76@%	19.10 ± 9.63#$%	< 0.001
LL (°)	45.21 ± 8.26!@#	33.46 ± 10.64!$	29.08 ± 11.52@%	13.36 ± 12.56#$%	< 0.001
TLK (°)	–6.57 ± 8.21	–8.01 ± 7.53	–6.50 ± 8.00	–6.49 ± 4.53	0.693
TK (°)	–36.57 ± 9.70!@#	–32.22 ± 11.48!	–28.57 ± 12.40@	–24.74 ± 8.91#	0.005
C7S (°)	25.42 ± 5.66!	27.13 ± 6.08@	27.11 ± 6.89#	34.11 ± 10.57!@#	0.008
C7-SVA (cm)	–0.9 ± 2.06!@#	3.18 ± 3.94!$%	7.32 ± 2.54@$^	12.14 ± 1.96#%^	< 0.001
ODI (%)	10.33 ± 6.78!@#	28.01 ± 16.61!	27.30 ± 11.19@	27.71 ± 12.67#	< 0.001
Lumbar VAS	3.12 ± 1.39!@#	5.63 ± 2.05!	4.96 ± 1.99@	5.57 ± 1.27#	< 0.001
Leg VAS	2.76 ± 1.87	2.77 ± 1.78	2.61 ± 1.80	4.00 ± 2.45	0.357

!,@,#,$,% and ^ indicated P-value of Fisher least significant difference post hoc test < 0.05

PT: pelvic tilt; SS: sacral slope; PI: pelvic incidence; TK: thoracic kyphosis; LL: lumbar lordosis; C7S: C7 slope; TLK: thoracolumbar kyphosis; C7-SVA: C7 sagittal vertical axis; ODI: Oswestry Disability Index; VAS: visual analog scale

We compared demographic data and radiological parameters among the four compensatory classification groups (Table 4) and found no significant differences in TLK and Leg VAS (P = 0.702 and P = 0.524, respectively). However, there were significant differences in age, gender, PI, PT, SS, LL, TK, C7S, C7-SVA, ODI, and Lumbar VAS (P < 0.001, P = 0.046, P < 0.001, P < 0.001, P = 0.009, P < 0.001, P < 0.001, P < 0.001, P < 0.001, P < 0.0.01, and P < 0.001, respectively). Imbalance without compensation patients were older, there were more males, and there was larger PT, less LL, larger C7S, larger C7-SVA, more ODI, and more Lumbar VAS than the Balance group (P = 0.037, P < 0.001, P < 0.001, P < 0.001, P < 0.001, P < 0.001). Imbalance with compensation patients had larger PI, larger PT, smaller SS, smaller TK, larger TK, larger C7-SVA, more ODI, and more Lumbar VAS than balance patients (P = 0.01, P < 0.001, P = 0.01, P < 0.001, P < 0.001, P < 0.001, and P < 0.001, respectively). The Hidden imbalance group had larger PI, larger PT, smaller SS, smaller LL, larger TK, larger C7-SVA, more ODI, and more Lumbar VAS than the Balance group (p = 0.002, P < 0.001, P = 0.016, P < 0.001, P = 0.002, P = 0.001, and P < 0.001, respectively).

**Table 4:** Comparison among the compensatory classification groups

	Balance (59)	Hidden imbalance (55)	Imbalance with compensation (36)	Imbalance without compensation (12)	P
Age(years)	62.05 ± 10.89!@	62.87 ± 10.98#$	68.69 ± 9.00!#%	76.25 ± 7.93@$%	< 0.001
Gender (M/F)	19/40!	14/41@	7/24#	10/7!@#	0.046
PI (°)	47.66 ± 8.03!@	52.91 ± 8.38!#	54.49 ± 9.83@$	46.88 ± 7.92#$	< 0.001
PT (°)	12.15 ± 5.73!@	21.27 ± 7.03!#$	24.85 ± 10.21@#%	13.67 ± 4.26$%	< 0.001
SS (°)	35.55 ± 8.42!@	31.68 ± 7.90!	29.63 ± 9.71@	33.22 ± 8.02	0.009
LL (°)	44.86 ± 8.83!@#	33.59 ± 9.30!$	24.03 ± 11.90@$%	34.92 ± 12.00#%	< 0.001
TLK (°)	-7.61 ± 8.24	-7.06 ± 7.77	-6.40 ± 7.31	-9.28 ± 5.84	0.702
TK (°)	-37.11 ± 9.56!@	-31.29 ± 10.62!#$	-25.13 ± 11.53@#%	-40.82 ± 7.30$%	< 0.001
C7S (°)	24.37 ± 5.13!	22.42 ± 5.54@#	26.09 ± 5.94@$	29.80 ± 4.77!#$	< 0.001
C7-SVA (cm)	-0.16 ± 2.53!@#	1.45 ± 2.54!$%	8.71 ± 2.84@$	8.14 ± 2.6#2%	< 0.001
ODI (%)	10.70 ± 7.12!@#	25.47 ± 15.35!$	28.94 ± 12.26@%	45.26 ± 11.61#$%	< 0.001
Lumbar VAS	3.28 ± 1.44!@#	5.05 ± 1.75!$	5.50 ± 1.84@%	8.17 ± 1.85#$%	< 0.001
Leg VAS	2.85 ± 1.86	2.64 ± 1.51	2.72 ± 2.17	3.50 ± 2.15	0.524

!,@,#,$,% and ^ indicated P-value of Fisher least significant difference post hoc test or Kruskal-Wallis H test < 0.05

PT: pelvic tilt; SS: sacral slope; PI: pelvic incidence; TK: thoracic kyphosis; LL: lumbar lordosis; C7S: C7 slope; TLK: thoracolumbar kyphosis; C7-SVA: C7 sagittal vertical axis; ODI: Oswestry Disability Index; VAS: visual analog scale

To identify risk factors for severe symptoms (ODI > 30%), stepwise regression analysis was performed using the dichotomous variable logistic regression model (Table 5). Higher compensatory classification was a significant risk factor for severe symptoms.

**Table 5:** Stepwise logistic regression for ODI > 30%

Variable	Coefficient of regression	Standard error	Wald x²	*P*	OR	95% confidence interval
Compensatory classification	1.323	0.241	30.070	< 0.001	3.755	2.340–6.025

ODI: Oswestry Disability Index

## Discussion

When standing, the center of gravity in the sagittal plane falls between the feet to ensure the balance of the sagittal plane of the spine and maintain the standing posture with minimum energy output. After spinal degeneration, lumbar disk degeneration and other factors cause the gradual reduction of LL and even lumbar kyphosis deformity. Only when other parts of the spine and pelvis compensate can the sagittal balance of the spine be maintained. However, when the spine cannot compensate for the loss of LL, the center of gravity of the spine moves forward to increase energy output and cause symptoms such as lumbar pain. For this reason, it is critical to evaluate spine-pelvis parameters for patients with low back pain [2, 3].

Takemitsu first proposed lumbar degenerative kyphosis in 1988. The author divided lumbar degenerative kyphosis into four types according to the degree and range of lumbar kyphosis and the maintenance of upright walking [8]. In recent years, investigators proposed that lumbar kyphosis be renamed primary degenerative sagittal imbalance; the diagnostic criteria were C7-SVA ≥ 5 cm, PI-LL ≥ 15°, and PT ≥ 25 ° [9]. Currently, the evaluation of adult spinal deformity uses the SRS-Schwab classification [7]. According to SRS-Schwab classification sagittal modifiers, the degree of spine sagittal deformity is assessed using PI-LL, C7-SVA, and PT.

In our clinic, we observed that some patients had increased PT but no significant increase in C7-SVA and some patients had increased C7-SVA but no significant pelvic supination compensation. There is a lack of understanding of the condition of such patients. Therefore, we grouped patients by spinal compensation mechanism to explore the relationship between spine-pelvis sagittal parameters and symptoms. We found that severe deformity patients had relatively severe symptoms using the SRS-Schwab classification. However, stepwise regression analysis did not identify SRS-Schwab classification as significantly correlated with a poor ODI.

Gille et al. created a detailed grouping of patients with lumbar spondylolisthesis according to the compensation of the lumbar spine and pelvic compensation [10]. Other studies divided the sagittal balance of the spine into the balanced spine, hidden unbalanced spine, and unbalanced spine [3, 11]. During spinal degeneration, compensation can temporarily maintain spinal balance. Only when losing compensation does the degenerative deformity of the spine cause significant imbalance and aggravation of symptoms.

**Fig. 4:**
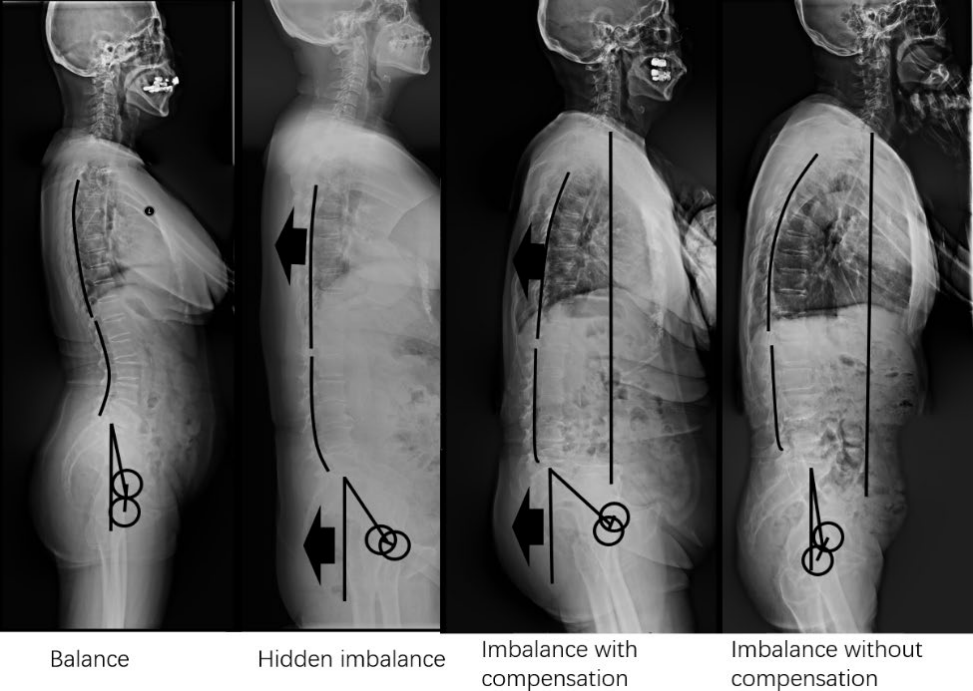
Four groups based on the compensatory classification

Using correlation analysis between symptom scores and spine-pelvis sagittal parameters, we found that ODI, Lumbar VAS, and Leg VAS correlated with LL. Greater LL loss correlated with more severe symptoms. However, SS correlated with ODI; TK and C7-SVA correlated with ODI and Lumbar VAS. These findings suggest that the degree of lumbar degeneration and sagittal balance of the spine are related to symptoms, consistent with previous studies of patients with reduced LL and sagittal imbalance [2, 5, 6, 12]. However, previous studies lacked discussion of patients with pelvic and thoracic compensation; therefore, the patients were divided into four groups according to the loss of LL and the compensation of the pelvis and thoracic spine. The matching of PI and LL was used to analyze whether lumbar lordosis was lost. PT > 20 ° suggested apparent pelvic posterior rotation compensation. TK > − 30° suggests thoracic extension compensation. In the present study, hidden imbalance and imbalance with compensation patients had a thoracic extension and pelvic rotation compensation, preventing the center of gravity from moving forward. Imbalance without compensation patients had similar TK and PT to balance patients, but severe symptoms (Fig. 4). Correlation analysis and stepwise regression showed that the symptoms of the four compensatory classification groups were aggravated.

No studies examine the reasons for the varying compensatory abilities among patients. Nevertheless, advanced age, muscle atrophy, and osteoarthritis may reduce the compensatory ability of the pelvic and thoracic spine [2, 13]. Therefore, elderly patients should be thoroughly evaluated before surgery to improve outcomes. The compensatory ability and thoracic flexibility should be considered for patients in different groups when planning surgery. Patients without compensation may need full correction of LL to achieve postoperative sagittal balance. Of course, lumbar procedures and efficacy assessments must be performed for patients in different groups. Nevertheless, the present study provides a theoretical basis for a preliminary evaluation of sagittal spinal balance.

Currently, the overall sagittal balance of the spine is evaluated using C7-SVA, which correlates with symptoms [5, 6]. However, in clinical application, the authors found that C7-SVA correlated with standing posture. One study used a patient with ankylosing spondylitis and found that different standing postures caused changes in SVA of 14.16 cm [14]. Patients with a sagittal spine imbalance may lean forward after standing and walking for long periods, giving rise to C7-SVA, which is more significant than in the resting state. These findings suggest many influencing factors in C7-SVA measurement, especially in older patients, who have a wide range of acceptance of C7-SVA [7]. It is challenging to evaluate the balance of the spine and pelvis accurately.

This study considered four groups to provide a basis for evaluating spine-pelvis sagittal balance and understanding the changes in spine-pelvis sagittal position after lumbar degeneration.

Miyagi et al. evaluated four SRS-Schwab classification groups of sagittal spinal malalignment, body mass index, grip strength, and trunk muscle mass [15]. They found that aging, obesity, low trunk muscle mass, and low grip strength were potential risk factors for sagittal spinal malalignment. Low trunk muscle mass might be one explanation for compensatory ability loss. Global alignment and proportion scores were used to predict mechanical complications [16]. However, global alignment and proportion focus on postoperative evaluation and cannot be widely used for preoperative surgical planning. More research related to spinal compensation is required.

Buckland et al. studied different compensatory behaviors in lumbar spinal stenosis and ASD [17]. Although the compensatory mechanism is different in lumbar spinal stenosis with mild deformity and ASD patients, compensatory mechanisms are similar between lumbar spinal stenosis with severe deformity and ASD patients. Although there are many reasons for spinal deformity, the compensatory mechanism seems to be the same after severe spinal deformity for upright posture.

Surgical planning for any surgical treatment of spine pathology should consider sagittal balance and compensation. In addition to good nerve decompression, maintaining good spinal balance is critical. The compensation of the deformity spine reflects the flexibility of the spine. When there is an imbalanced spine with compensation, local correction surgery might be able to maintain spine sagittal balance. When imbalance without compensation, osteotomy, and long-segment fusion surgery to achieve sufficient correction might be necessary. Of course, all correction surgery plans need to consider the situation of nerve compression. The outcome is based on good decompression and correction.

There are some limitations to this study. Because the sample size was relatively small, it was impossible to create more detailed groupings in terms of compensation. The basic pelvic morphology was not grouped using the PI or Roussouly classification; we divided patients into only four groups for comparison. Another limitation was that we grouped patients based on radiographs alone (i.e., no magnetic resonance imaging). This is a substantial limitation that does not allow surgeons to assess the reasons for spine deformity or evaluate neurogenic claudication or other neural element compression and their effects on VAS and ODI scores. Therefore, we will need to combine MRI and full-length X-ray for grouping in future studies. In addition, spinal degeneration is a complex process, and the change of LL, muscle atrophy, bone loss, and arthritis might affect spinal morphology. There is a lack of research evaluating the compensatory ability of the thoracic spine and pelvis, and studies are needed to overcome these limitations. Only patients with LL loss were included in this study; sagittal imbalance patients who resulted from thoracolumbar kyphosis were not studied. Nevertheless, from the perspective of pelvic compensation and thoracic compensation, this study analyzed the value of compensation in the sagittal balance of spine-pelvis in patients with lumbar degeneration, which might have clinical significance.

## Conclusion

Based on the grouping of pelvic and thoracic compensation, the evaluation of spinal-pelvic sagittal balance in patients with lumbar degeneration can reflect the spinal balance and symptoms and avoid the center of gravity’s inaccurate evaluation of spinal balance. This paradigm might be helpful for elderly patients with lumbar degeneration. Sufficient correction surgery may be necessary for imbalanced patients without compensation.

## Data Availability

The datasets used and/or analysed during the current study are available from the corresponding author upon reasonable request.
